# Cardiac-Targeting Peptide: From Discovery to Applications

**DOI:** 10.3390/biom13121690

**Published:** 2023-11-23

**Authors:** Daniella Sahagun, Maliha Zahid

**Affiliations:** Department of Cardiovascular Medicine, Mayo Clinic, Guggenheim Gu9-01B, Mayo Clinic, 200 First St. SW, Rochester, MN 55905, USA; sahagun.daniella@mayo.edu

**Keywords:** cell-penetrating peptides, protein transduction domains, cardiac-targeting peptide, heart failure, atrial fibrillation

## Abstract

Despite significant strides in prevention, diagnosis, and treatment, cardiovascular diseases remain the number one cause of mortality in the United States, with rates climbing at an alarming rate in the developing world. Targeted delivery of therapeutics to the heart has been a lofty goal to achieve with strategies ranging from direct intra-cardiac or intra-pericardial delivery, intra-coronary infusion, to adenoviral, lentiviral, and adeno-associated viral vectors which have preference, if not complete cardio-selectivity, for cardiac tissue. Cell-penetrating peptides (CPP) are 5–30-amino-acid-long peptides that are able to breach cell membrane barriers while carrying cargoes up to several times their size, in an intact functional form. Identified nearly three decades ago, the first of these CPPs came from the HIV coat protein transactivator of transcription. Although a highly efficient CPP, its clinical utility is limited by its robust ability to cross any cell membrane barrier, including crossing the blood–brain barrier and transducing neuronal tissue non-specifically. Several strategies have been utilized to identify cell- or tissue-specific CPPs, one of which is phage display. Using this latter technique, we identified a cardiomyocyte-targeting peptide (CTP) more than a decade ago, a finding that has been corroborated by several independent labs across the world that have utilized CTP for a myriad of different purposes in pre-clinical animal models. The goal of this publication is to provide a comprehensive review of the identification, validation, and application of CTP, and outline its potential in diagnostic and therapeutic applications especially in the field of targeted RNA interference.

## 1. Introduction

Despite major advances in the diagnosis and treatment of cardiovascular disorders, they remain the number one killer in the developed world, with rates rising at an alarming rate in the developing world [1,2,3,4]. A major cardiovascular pathology is atrial fibrillation; the most common rhythm disorder in adults with an estimated prevalence of 46.3 million worldwide [5]. It affects 1–2% of adults in the United States, with an estimated 5.7 million patients [6]. Data from the Framingham heart study have documented an over threefold increase in its prevalence over the last 50 years [7]. These already-high prevalence rates are further estimated to double by year 2050 due to an aging of the population, and increased prevalence of risk factors like coronary artery disease, hypertension, obesity, and congestive heart failure [7]. Superiority of rhythm over rate control in this disorder has been demonstrated in multiple randomized control trials [8,9,10]. Cardiomyocytes, the cells responsible for the contractility of the heart, are the seat of some common pathologies like heart failure with reduced ejection fraction due to a myriad of different reasons. Another type of heart failure, heart failure with preserved ejection fraction, results from not a “weak” but a “stiff” heart, and has been rising steadily with the proportion of all heart failure patients, due to the aging of the population, and increasing incidence of co-morbidities, like obesity, hypertension, and diabetes mellitus that lead to it. In stark contrast to heart failure with reduced ejection fraction, heart failure with preserved ejection fraction is a recalcitrant disease with very few therapies shown to benefit it. Targeting the stiffened heart by changing the behavior of the hypertrophied cardiomyocyte, hence addressing the underlying pathophysiology, would require the ability to successfully and specifically target the offending cardiomyocyte.

In our era of advanced cardiac diagnostics and a multitude of therapeutics available to the clinician, targeted drug delivery is the next frontier [11]. It is increasingly being recognized that designing new drugs is not enough; they have to be delivered to a specific organ, and preferably to the cells of interest. In the context of targeting the heart, the strategies have ranged from physical approaches ranging from direct microneedle injections into the myocardium [12,13], intra-coronary instillation [13], retrograde perfusion of the coronary sinus [14,15], and placement of hydrogels into the pericardial space [16,17,18,19]. On the vector side, viral vectors have ranged from adenovirus [20,21], lentivirus [22], adeno-associated viruses [23,24], or non-viral vectors comprising exosomes [25], nanoparticles [26,27] or microbubbles targeted to the heart by destruction of these bubbles with ultrasound during their passage through the heart under direct visualization [28,29,30]. An alternate, non-viral, promising strategy is the use of cell-penetrating peptides (CPPs), also known as protein transduction domains [31].

First identified nearly three decades ago, CPPs are 5–30-amino-acid-long peptides that are able to cross cell membrane barriers while carrying cargoes several times their size in an intact, functional form. As so often happens in science, this was a serendipitous discovery. Two independent groups of scientists investigating the various functions of the HIV coat protein transactivator of transduction (Tat), an 86-amino-acid protein, discovered the protein’s ability to enter cells efficiently without the use of transfection reagents [32,33]. Soon to follow was the report that homeobox domain of antennapedia transcription factor of Drosophila melanogaster was able to enter neural cells in a receptor-independent manner and affect neural morphogenesis [34]. Mapping of the domains within Tat responsible for its transduction property led to the identification of a positively charged segment of the protein, only 11 amino acids long, rich in arginine and lysine that are two cationic amino acid residues (YGRKKRRQRRR) [35]. Subsequently, the ability to carry cargoes much larger than its size was demonstrated by fusing Tat to a beta-galactosidase reporter protein residue, which upon intra-peritoneal injection into wild-type mice led to robust transduction of multiple tissues, including heart, lung, liver, kidneys and brain tissue, at six hours post injection [36], highlighting Tat’s potential as a vector. However, this robust, ubiquitous uptake by myriad tissue, especially crossing of the blood–brain barrier, limited its clinical utility. Researchers have tackled this issue by using Tat locally instead of systemically, by intra-articular, intra-ocular or intra-tumoral injections. This approach is not feasible for the targeting of deeper organs such as the heart due to its invasiveness and resulting complications, which would limit repeat administrations.

Since the initial description of the first two CPPs, Tat and Antennapedia homeodomain, the family of CPPs has expanded exponentially, as has the number of publications related to them. Non-specific uptake of these peptides has been tackled by either use of localized delivery, or administration of peptides that are only activated for uptake in specific environments, like tumor micro-environments, or use of phage delivery to identify tissue specific CPPs. The concept of phage display was first proposed by G. P. Smith in 1985 [37] in a single-author Science publication, where he demonstrated that foreign DNA could be inserted into filamentous phage gene III to create fusion protein that would display the inserted amino acids on the surface protein where it could be enriched over 1000-fold. Since the initial report, combinatorial peptide libraries of various lengths utilizing different filamentous phage (M13, T7), displaying millions of different, randomized amino acid sequences have been utilized in phage display to identify peptides homing to various targets in vitro and in vivo. We utilized a combinatorial in vitro and in vivo phage display methodology [38] to identify a 12-amino acid long, non-naturally occurring peptide that specifically targets cardiomyocytes [39], which we termed cardiac-targeting peptide (CTP) and forms the subject of the rest of this review. Studies highlighted in this review are peer-reviewed and span our work as well as the work of other investigators from around the world published only in indexed medical journals available on PubMed.

## 2. Identification of Cardiac-Targeting Peptide

We utilized a combinatorial in vitro and in vivo phage screening of a large, commercially available M13 phage display library to identify CTP, a cardiomyocyte-specific cell-penetrating peptide [39]. The heart is a very vascular organ with only a small minority of the intravascularly injected phage being internalized, with the blood pool contaminating what is recovered from the cardiomyocytes themselves. To enrich for cardiac-targeting peptides, we attempted to decrease non-specific phage by pre-screening in a rat cardiomyoblast, H9C2, cell line. Cells were plated in a six-well plate and allowed to reach 70% confluency before being incubated with the phage library for 6 hrs. At the end of the incubation period, cells were washed extensively, trypsinized, collected and lysed using freeze–thaw cycles. Phage recovered from the lysed cell was expanded, injected into a mouse and allowed to circulate for 24 h. The rationale for prolonged circulation time was to allow for non-specific phage from the blood pool to clear out [40], allowing for only internalized phage to be recovered from heart tissue. In order to maximize viability of the internalized phage, mice were treated 24 h before the injections and on the day of the injection with Chloroquine to prevent acidification of lysosomes and resulting breakdown of internalized phage. Recovered phage from the heart was expanded and reinjected for subsequent cycles. After the 4th cycle, 10 plaques were picked and sent for sequencing, with 6 out of 10 bearing the identical sequence of APWHLSSQYSRT, a peptide that we later named CTP or cardiac-targeting peptide. In vitro incubation of a number of different cell lines with fluorescently tagged CTP revealed a preferential uptake of the peptide by H9C2 cells [39]. Injecting mice with fluorescently tagged CTP showed robust uptake by heart in 30 min. Later biodistribution studies revealed peak uptake at 15 min (earliest time point tested) with almost complete disappearance of fluorescence by 6 h [41].

Phage display had been utilized prior to the above experiments in an in vitro display methodology to identify another, longer cardiomyocyte-homing peptide. McGuire and colleagues used an in vitro phage display approach to identify a 20-mer amino acid (WLSEAGPVVTVRALRGTGSW) which had partial sequence homology to tenascin-X, an extracellular matrix protein [42]. This peptide had a 180-fold preference for cardiomyocytes in vitro, the cell line used for phage display. They were able to show preferential isolation of this phage from heart tissue as compared to a random peptide-bearing phage [42]. More recently, an independent group were able to engineer cardiosphere-derived cells to produce exosomes labeled with Lamp2b and WLSEAGPVVTVRALRGTGSW, which showed anti-apoptotic effects in vitro in primary mouse neonatal cardiomyocytes, and increased cardiac retention in vivo compared with non-labeled exosomes [43]. However, it is worth noting that all in vivo injections into mice were intramyocardial, and not systemic or intravenous, bringing into question the strength of the targeting as well as the clinical applicability of this approach for use in humans. This is an important point as intra-myocardial delivery is clinically feasible in a very limited number of patients such as those undergoing coronary artery bypass grafting or another open-heart procedure, precluding repeat therapies. In another application, L-arginine-loaded gold nanoparticles modified with WLSEAGPVVTVRALRGTGSW exhibited increased cellular uptake by cardiomyocytes with resulting improved photoacoustic imaging in vitro and in vivo [44]. These nanoparticles were able to increase nitric oxide production, and decrease apoptosis, fibrosis and infarct size in a rat model of ischemia–reperfusion [44]. Hopefully, larger animal studies will be forthcoming and show similar efficacy, as this was a very recent publication.

## 3. Cardiac-Targeting Peptide for Diagnostic Purposes

**Figure 1:**
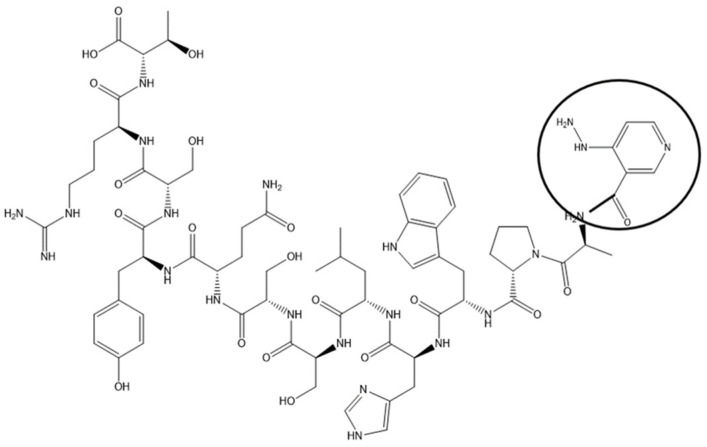
Chemical Structure of CTP-HYNIC Conjugate. An example of imaging application of CTP by synthetizing it with HYNIC conjugated to the N-terminus and labeled with ^99m^Tc-HYNIC-CTP. Wild-type CD1 mice were injected with 1–5 mCi of ^99m^Tc Sestamibi or ^99m^Tc-HYNIC-CTP, and single-photon-emission computed tomography (SPECT/CT) imaging performed [42].

Coronary artery disease is the most common form of cardiovascular disorder. Atherosclerotic plaque buildup leads to progressive narrowing of coronary artery lumen. This is a chronic process, with symptoms developing only after a significant portion of the lumen (>50–70%) has been compromised. Symptoms, once they develop, range from chest pain, shortness of breath on exertion, to fatigue and decreased exercise tolerance, and are responsible for the most frequent reason for presentation to the emergency rooms in the US. These symptoms are investigated in patients by putting them through cardiac stress testing while continuously monitoring the heart with electrocardiograms (ECGs). The heart is stressed with exercise, the most physiological form of stressor, or chemicals in patients who cannot exercise. Commonly, ECG alone has low sensitivity and specificity for detection of occlusive coronary artery disease, and hence is combined with an imaging modality, in the form of nuclear cardiac imaging or echocardiography using ultrasound. Myocardial perfusion imaging is most commonly performed through using cardiac tracers like Thallium, or, more recently, Technetium 99^m^. Uptake of this tracer by the liver and gut loops interferes with heart imaging, lowering the sensitivity of the examination. Targeting the radioisotopes to the heart would, in theory, improve cardiac uptake and decrease radio-isotope dose. CTP was tagged with Technetium 99^m^ via a chelator, HYNIC (Figure 1), and showed almost exclusive uptake by the heart with predominantly renal excretion, when compared with Technetium Sestamibi, the formulation of Technetium 99^m^ in wide-spread clinical use [41].

Cardiac PET imaging is a more recent addition to the clinician armamentarium of diagnostics. Compared to SPECT imaging, PET has a higher sensitivity (80.3% versus 68.7%) and similar specificity (63.8% versus 61.7%) and therefore overall superior accuracy in predicting occlusive coronary artery disease with significantly lower radiation exposure (by ~50%) than SPECT. Yet, it remains grossly under-utilized with data from 2019, indicating that of all nuclear cardiac stress tests, PET studies represented only 10% of them. The underlying reason for this is lack of widespread availability of cardiac PET tracers. The only FDA-approved cardiac PET tracers in clinical use are ^15^O water, ^13^N ammonia, and ^82^Rubidium [13,14]. The availability of these tracers is limited by the need for an on-site (^15^O water and ^13^N ammonia) or nearby (^13^N ammonia) cyclotron, or commitment to costly generators (^82^Rb). Due to the short half-lives ranging from 76 s for ^82^Rb, to 2.1 min for ^15^O water and 10 min for ^13^N ammonia, their use in conjunction with treadmill exercise stress testing is either not possible (^82^Rb and ^15^O water) or not practical (^13^N ammonia). Furthermore, the long positron range of ^82^Rb makes image resolution suboptimal and its low extraction limits its spatial resolution. Of all stress modalities (exercise, dobutamine, dipyridamole, adenosine, and regadenoson), exercise is the most physiological stressor, and provides important additive prognostic information not available with other stressors.

With this background in mind, CTP could provide a solution. Our recent work showed that labeling of CTP with Gallium-68, another PET radioisotope, can be successfully performed using 1,4,7-Triazacyclononane-1,4,7-triacetic acid (NOTA) as a chelator, with preliminary animal studies showing promise. This conjugation was optimized to yield 98% radiolabeling efficiency at room temperature in ten minutes, and with >97% radiochemical purity, without the need of a heating block. The CTP-NOTA-Gallium 68 conjugate was taken up by heart tissue in as little as 5 min with isotope leaving the heart in ~30 min. At later time points, the gall bladder lit up on PET imaging, with a significant portion being secreted by the kidneys (presented in abstract form at Society of Nuclear Medicine, Chicago, 2023).

## 4. Cardiac-Targeting Peptide for Therapeutic Purposes

The first therapeutic application of CTP was studied by Avula and colleagues in order to perform cell-selective arrhythmia ablation [45]. They engineered an 8-arm pegylated nanoparticle with an average of 2.6 CTP molecules attached to the pegylated arms along with a photosensitizer chlorin e6. Injecting these nanoparticles into rats followed by laser illumination of the heart induced localized myocyte-specific ablation restoring normal rhythm with 85% efficiency. Of note, there was no damage to “innocent, bystander” cells like endothelial cells or myofibroblasts. They were also able to demonstrate this cardiomyocyte-specific ablation in sheep heart ex vivo. This led to the formation of complete heart block at the ablated region leading to restoration of normal sinus rhythm [45].

Extracellular vesicles, or exosomes, are heterogeneous, membrane bound vesicles, ranging in size from nanometers to micrometers, and originate as extrusions from the endosome or plasma membrane. They are being actively explored by a number of researchers as delivery vehicles for cardiac and other applications. In a series of experiments, researchers from South Korea were able to engineer exosomes expressing CTP along with LampB, and showed ~15% increased uptake in vitro and in vivo compared to non-targeted (LampB but no CTP bearing) exosomes [46]. In another demonstration of this targeted delivery, exosomes labeled only with CTP were loaded with curcumin and miR-144-3p, and showed not only in vivo delivery to the murine heart, but also cardioprotection in a mouse infarct model [47]. In a separate application, Kim and colleagues engineered extracellular vesicles to express high levels of CTP [48]. These vesicles were loaded with anti-RAGE (receptor for advanced glycation end products) siRNA. RAGE has been shown to contribute to inflammation in a number of cardiac pathologies that lead to myocarditis. Such labeled vesicles were injected intravenously into a rat model of myocarditis induced by immunization with cardiac myosin. On day 7 after induction of myocarditis, rats were treated with a single injection of non-targeted vesicles loaded with anti-RAGE siRNA or targeted vesicles labeled with CTP. Targeted vesicles significantly decrease RAGE, IL-6, and TNF-α levels in heart tissue. This decrease in multiple inflammatory markers was accompanied by a significant increase in left ventricular ejection fraction, and improvements in left ventricular end-systolic and end-diastolic dimensions [48]. Treatment also led to a significant decrease in protein levels of RAGE (as expected), but also levels of Il-6, TNF-α, COX2 and proportion of phosphorylated p65, an activated component of the NF-κappa-B pathway [49].

As stated earlier, heart failure with preserved ejection fraction is growing in incidence as well as a disease particularly recalcitrant to treatments by drugs and interventions that have been shown to be beneficial in heart failure with reduced ejection fraction. This is likely due to significant differences in underlying pathophysiology. One of the hallmarks of heart failure with preserved ejection fraction is a thick hypertrophied left ventricle with increased myocyte stiffness. To target this particular pathology at the cardiomyocyte level, Gallicano and colleagues worked to deliver microRNA106a to the cardiomyocyte. MicroRNAs are small non-coding, ~22-nucleatide-long RNAs that silence gene expression via a post-translation modification of mRNAs. They target the 3′UTR of an mRNA through a 7–8 bases long seed sequence that marks the complex for degradation in the RISC or RNA-induced silencing complex. One of the targets of miRNA106a is calcium calmodulin kinase IIδ that is upregulated in heart failure and responsible for the calcium mishandling intrinsic to these hypertrophied cardiomyocytes. In order to deliver miRNA106a specifically to hypertrophied cardiomyocytes, it was conjugated to the N-terminus of CTP via a disulfide linker. This linker was chosen due to its covalent nature and high likelihood of remaining stable in serum, as well as being broken down in the reducing intracellular environment, leading to release of cargo miRNA106a from its vector CTP, allowing it to bind to calcium calmodulin kinase Iiδ mRNA marking it for degradation in the RISC complex. Incubation of human left ventricular myocytes with fluorescently labeled CTP-miRNA106a conjugate led to robust uptake by the cells [50]. This conjugate was selectively taken up by human cardiomyocyte cell line and not by HEK293 cells [50]. Additionally, this conjugate was able to reverse angiotensin–phenylephrine-induced hypertrophy of myocytes by decreasing expression of both HDAC4 and calcium calmodulin kinase IIδ, both targets of miRNA106a [49]. Studies on the effect of this conjugate in reversing cardiac hypertrophy and ameliorating heart failure in an in vivo mouse model are ongoing.

In our own work, we chose to utilize CTP to deliver amiodarone, a small molecule class III anti-arrhythmic. Amiodarone was approved by the FDA for use as an antiarrhythmic for ventricular tachycardia and fibrillation, two life-threatening rhythms, in December 1985. A frequent off-label use of it has been for atrial fibrillation, a common nuisance rhythm that puts patients at increased risk of congestive heart failure as well as strokes. In fact, as many as 20–25% of strokes in the US are secondary to clot formation in the heart resulting from atrial fibrillation. In spite of amiodarone being the most effective anti-arrhythmic, its use is limited due to its vast volume of distribution in the human body, prolonged half-life, with only a small fraction of it (<5%) going to the heart. There are also significant, and sometimes life-threatening, off-target toxicities associated with it. Our hypothesis was that by using CTP as a vector, we can direct amiodarone to the heart where it is needed to act, thereby reducing the total dose needed to see the same anti-arrhythmic effects and decreasing off-target uptake, hence improving its safety profile. To that end, we conjugated amiodarone to the N-terminus of CTP via a disulfide bond [50]. Using high-pressure liquid chromatography, we showed that this conjugate is stable at 37 °C for up to 22 days [50]. We tested this conjugate in vivo in guinea pigs that were injected with either vehicle, amiodarone (80 mg/kg for 7 days), CTP-amiodarone, or CTP, with the last two (CTP-amio and CTP) injected at 1/10th the molar dose of amiodarone, for 5 days [51]. At the end of the treatment period, guinea pigs were euthanized, hearts excised and perfused in a Langendorf apparatus. Voltage and calcium indicator dyes were injected and fluorescence from the epicardium of the heart split and focused onto two cameras to record cytosolic calcium currents and voltage conduction velocities. Amiodarone, as per expectations, slowed the heart rate, and decreased calcium conduction and voltage conduction velocities, hence increasing the action potential durations. CTP-amiodarone also decreased calcium and voltage conduction velocities, similar to amiodarone. To our surprise, CTP-amiodarone increased the heart rate, decreased action potential duration and decreased late calcium transients, effects that seem to be driven by the CTP portion of the conjugate. In order to explore the underlying possible mechanisms for these observations, RNA was extracted from hearts, RNA sequencing was performed, and the results of the CTP-injected hearts were compared to control (vehicle-injection-only) hearts. Two important calcium handling genes, SERCA2a and DHPR, were upregulated in CTP-injected hearts, as were alpha adrenergic receptors. On the other hand, beta adrenergic receptors were downregulated, explaining the significant increase in heart rates. Most interestingly, several inflammatory genes in the NF-kappa B pathway, like TNFα, IL1β, and COX2 were significantly downregulated in the CTP-treated hearts [51]. These were unexpected findings as we had considered CTP to always be an inert vector. Whether CTP goes beyond being a vector and has anti-inflammatory effects and salutary effects on calcium handling remains to be confirmed, and studies exploring these intriguing but preliminary findings are underway.

## 5. Challenges, Limitations and Future Studies

Since the report of its initial discovery in 2010 [39], the literature surrounding CTP has grown with a number of laboratories independently verifying its cardiomyocyte-specific targeting abilities after systemic delivery. Not only is it a “cardiac” targeting peptide, but it is also a cardiomyocyte-specific targeting peptide with possibility for cardiomyocyte physiology altering potential as demonstrated in work by Gallicano and colleagues [50]. Additionally, the number of cargoes delivered using CTP continue to grow. Yet, many unanswered questions remain, the foremost being its mechanism of transduction that is instrumental in targeting cardiomyocytes. In spite of multiple efforts, which remains an enigma. Cardiomyocytes are constantly beating cells with cell-to-cell connections, sodium–potassium channels as well as calcium channels. However, these unique proteins form small pores meant for ions that are logarithmic degrees of magnitude smaller in size, compared with CTP with or without a cargo.

Another important question that requires further study is toxicity. Our own data, generated in our lab in a non-good laboratory practices (non-GLP) environment, has shown no overt toxicities. However, our studies were not comprehensive or all-encompassing, and only tested CTP by itself (and a chelator, HYNIC, added), without a cargo. Moreover, these toxicity studies were designed for micro-dose, diagnostic studies for human application, and were hence single dose. To study the full genotoxicity profile of CTP and its cargo, multi-dose, longitudinal, longer-term studies in at least two vertebrate species (of which only one can be a rodent animal model) need to be undertaken. Due to the need for a GLP environment, such studies are typically undertaken by independent, third-party, certified research organizations and are costly. Lastly, CTP is platform technology, a non-viral method of delivering diagnostic agents and therapeutics to the heart, with many potential cargoes that could be delivered. To fully realize its potential will require concerted, and costly, efforts from multiple independent investigators.

## 6. Conclusions

CTP belongs to the larger class of tissue specific CPPs identified through phage display utilizing a commercially available phage display library. Uptake by the heart occurs in as little as 5–15 min, making it ideal for time-sensitive conditions like myocardial infarction, also known as “heart attacks” in layman terms and ischemia–reperfusion injuries. During a myocardial infarction, cardiomyocytes are suffering necrosis due to a blocked artery with a window of opportunity to intervene limited to 6 h or less. This window is far shorter than the reach of viral or plasmid vectors, or direct intramyocardial injections. The ability of CTP to target cardiomyocytes with peak uptake in as little as 5–15 min (depending on cargo size) adds to its utility as a vector. Furthermore, it is cardiomyocyte-specific, sparing other cells in the heart like myofibroblasts, endothelial cells or resident macrophages, an immensely valuable property in conditions like heart failure where cardiomyocytes are the seat of the pathology. Although many proof-of-principle studies in vitro and in vivo in small animal models have been carried out, studies in larger animals are lacking. Additionally, full toxicity studies of CTP with its cargo(s) of choice need to be carried out in order to allow human trials leading to clinical applications, and ultimate realization of its full potential.
